# Development of a clinical decision support system for breast cancer detection using ensemble deep learning

**DOI:** 10.1038/s41598-025-06784-2

**Published:** 2025-07-18

**Authors:** Jasjeet Kaur Sandhu, Chetna Sharma, Amandeep Kaur, Saroj Kumar Pandey, Anurag Sinha, J. Shreyas

**Affiliations:** 1 Chitkara University Institute of Engineering and Technology, Chitkara University, Rajpura, Punjab India; 2 Department of Computer Engineering & Applications, GLA University, Mathura, India; 3 Tech School, Computer Science Department, ICFAI University, Ranchi, Jharkhand India; 4 Department of Information Technology, Manipal Institute of Technology Bengaluru, Manipal Academy of Higher Education, Manipal, India

**Keywords:** Breast cancer, Deep learning, EDL-CDSS, Deep belief network, KELM, Cancer, Computational biology and bioinformatics, Health care

## Abstract

Advancements in diagnostic technology are required to improve patient outcomes and facilitate early diagnosis, as breast cancer is a substantial global health concern. This research discusses the creation of a unique Deep Learning (DL) Ensemble Deep Learning based on a Clinical Decision Support System (EDL-CDSS) that enables the precise and expeditious diagnosis of breast cancer. Numerous DL models are combined in the proposed EDL-CDSS to create an ensemble method that optimizes the advantages and reduces the disadvantages of individual techniques. The team improves its capacity to extricate intricate patterns and features from medical imaging data by incorporating the Kelm Extreme Learning Machine (KELM), Deep Belief Network (DBN), and other DL architectures. Comprehensive testing has been conducted across various datasets to assess the efficacy of this system in comparison to individual DL models and traditional diagnostic methods. Among other objectives, the evaluation prioritizes precision, sensitivity, specificity, F1-score, accuracy, and overall accuracy to mitigate false positives and negatives. The experiment’s conclusion exhibits a remarkable accuracy of 96.14% in comparison to prior advanced methodologies.

## Introduction

Cancer is known to be one of the major global causes of death^1,2^. Furthermore, cancer of the breast is a frequent affliction among women and a leading reason for cancer-related mortality worldwide. Research indicates that early identification of breast cancer enhances the patient’s general state of life and elevates survival rates. Individuals with this disease exhibit a reduced mortality risk^3^. Ultrasonography is a technology that is often used in the process of diagnosing breast cancer due to its ease of use, lack of associated discomfort, and high level of effectiveness in real-time^4^.

Breast cancer has the potential to produce fast metastasis, leading to the main tumor aggressively spreading breast cancer cells to distant organs such as the bone, liver, lung, and brain^5^. The high proportion of breast cancer that cannot be cured is mostly attributable to the disease’s metastatic characteristics. Though there seems to be a decline in the death rates associated with breast cancer, younger age groups are still considered high risk and have a low survival rate. A significant factor in preventing the fast advancement of breast cancer, in addition to the development of preventive methods, is the early detection of people who have breast cancer^6^. Table 1 depicts the patterns of osseous malignancies common in women with the correlating percentages.

**Table 1:** The most common female cancers^7^.

Different types of cancers in women	Percentage (%)
Leukemia	4
Lung and Bronchus	26
Liver and intrahepatic bile duct	2
Pancreas	7
Brain/Other nervous systems	2
Non-Hodgkin Lymphoma	3
Colon and Rectum	9
Uterine corpus	3
Breast	14
Ovary	5

The implementation of the EDL-CDSS system brings about the most notable improvement in breast cancer detection since it allows an earlier diagnosis to be made with more precision and a quicker turnaround time. DL techniques are employed by these systems to qualitatively evaluate medical images of breasts. The fast growth of machine learning, especially DL, has generated interest in using these approaches to improve cancer screening accuracy since early identification of breast cancer could save lives^8^. According to^9^, the use of DL models can enhance diagnostic accuracy and ease the workload of health practitioners by producing more specific and granular information from cancer images. This contrasts with other approaches that have used a majority voting mechanism, ensemble classification mechanisms, or DL algorithms specifically designed for detecting BC in mammograms with great results. These novel techniques can boost the precision of the diagnosis of BC, ultimately resulting in better patient outcomes^10,11^.

This paper describes the EDL-CDSS for the Breast Ultrasound Dataset benchmark from the perspective of the Internet of Things (IoT). This study also incorporates an ensemble of the two models, DBN as well as KELM. Furthermore, the hyperparameter optimization of the DBN model, as well as the KELM model is conducted using the Arithmetic Optimization approach. An exhaustive experimental investigation is carried out to investigate whether the EDL-CDSS method yields enhanced dataset results.

### Preventions for breast cancer

Research into breast cancer, both in the clinic and in the lab, has come a long way in recent years. In comparison to previous methods, screening, chemotherapy, as well as biological prophylaxis are significantly more effective in treating patients. Although the mortality rate from breast cancer has decreased, it continues to be the most prevalent reason for cancer-related fatalities among women aged 20 to 59.

### Breast cancer screening techniques

**Fig. 1:**
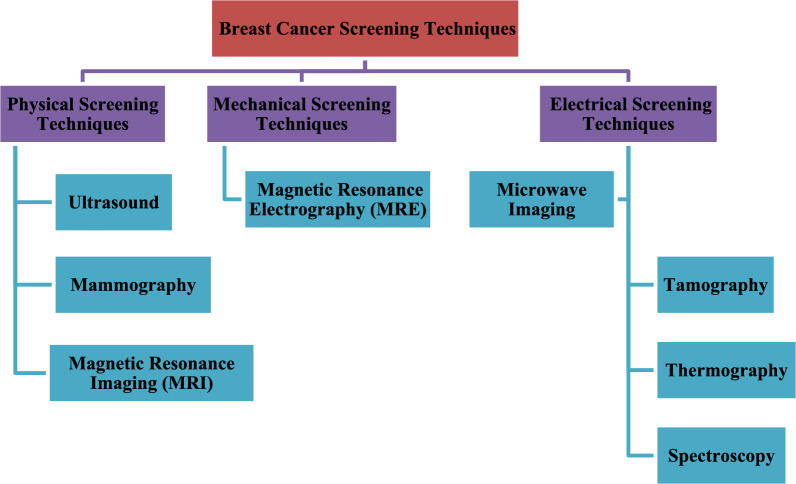
Breast cancer screening techniques categorized^12^.

Over 90% of cancer fatalities are due to metastases from secondary tumors, not primary tumors. When breast cancer is undetected metastatically or diagnosed at primary stages, it can surgically be removed, and treatment such as chemotherapy can help. Preventing breast cancer from occurring relies heavily on early detection. Figure 1 shows the categorization of various Breast Cancer Screening Techniques. In Fig. 1, many methods that aid in detecting breast cancer are illustrated and explained.

#### Physical screening techniques

Ultrasounds, mammograms and MRIs are the most frequently employed surveillance methods. Many techniques are elaborately defined in the subsequent subsections:

- **Mammography**

It is an X-ray examination of the breast that has the potential to detect either benign or malignant growth. The compressed breasts are subjected to a limited quantity of radiation between two plates to generate an X-ray image. Screening and diagnostic use for mammograms are possible^13^. Screening mammograms are used to look for breast cancer before any symptoms appear, with the hopes of reducing deaths caused by the disease. Breast cancer risk assessments could be enhanced by mammographic density. In addition, a DL model trained on mammograms could enhance risk prediction accuracy^14^.

- **Magnetic resonance imaging (MRI)**

MRI is a valuable contribution to the current breast cancer surveillance protocols. Breast MRI is a non-invasive procedure that employs a magnetic field and brief exposure to radiofrequency radiation to produce precise images of the breast’s interior^15^. Women who already have breast cancer can use MRI imaging to monitor for secondary tumors and determine their sizes. MRI is a better tool for exotic duct carcinoma in pregnancy when juxtaposed with mammography in severe duct carcinoma patients^16^. The insensitivity of MRI to breast density makes it more effective in detecting axillary nodal metastases, residual breast tumors post-neoadjuvant treatment, as well as other microtumors, making it superior to mammography in the identification of occult primary breast cancer.

#### Electrical screening techniques

Electrical screening is often carried out using tomography, transillumination, microwave imaging, thermography, and impedance spectroscopy. Certain approaches are further upon in the following sections:

- **Thermography**

A specialized camera is employed in thermography to record the surface skin temperature of the breast region. Thermography is the use of infrared radiation to determine an object’s internal temperature. It is a non-invasive, non-intrusive, passive, and radiation-free approach in contrast to other modalities^17^. The skin’s surface temperature reveals numerous aspects when used in medicine because the brightness of human skin is an exponential function of its surface temperature; that is, it is impacted by the amount of blood circulation in the skin^18^.

- **Microwave imaging**

A possible method of detecting breast cancer early may lie in microwave imaging. The fundamental driving force was the idea that the permittivity and conductivity of tissue from malignant breast tumors differed dramatically from those of the surrounding normal breast tissue. It was calculated that the gap was about five to 10 times greater. Additionally, microwave frequencies demonstrate acceptable penetration in breast tissue and are nonionizing but with low to moderate resolution. Transmitter and receiver antennas serve as sensors in microwave imaging^19^.

#### Mechanical screening techniques

The mechanical screening method known as Magnetic Resonance Elastography (MRE) is widely used nowadays. The next part provides a more in-depth explanation of this method.

- **Magnetic resonance elastography (MRE)**

The alterations in the viscoelastic properties of breast tissues under external stress can be evaluated using the breast MRE technique. Research evaluating the effectiveness of MRE for breast tissues remains in its infancy, leading to regular revisions and refinements of technology. The tumors of breast carcinomas have been demonstrated to have a high stiffness compared to surrounding benign tissues and are exploited via the use of tissue stiffness measurements^20^.

The novelty of this research lies in the development of an EDL-CDSS System that integrates SqueezeNet for efficient feature extraction with KELM and DBN classifiers for accurate diagnosis of breast cancer using ultrasound images. Unlike traditional models, this framework combines the strengths of lightweight convolutional architectures and robust classification techniques, enhanced further by hyperparameter tuning through the Arithmetic Optimization Algorithm. This integrated approach not only improves diagnostic accuracy and computational efficiency but also reduces false positives and negatives, offering a reliable, real-time, and scalable solution for early breast cancer detection in clinical settings.

## Related works

This section presented related works of various authors that are centered around the prediction of breast cancer employing DL techniques. The information includes the researcher’s name, the year of publication, the data sets used, the techniques employed, the domain targeted, the instruments used, and the key performance indicators (KPIs). Zakareya et al.^21^ suggested a new model for automated DL-based breast cancer diagnosis, which further improves classification accuracy. The comparison of the proposed framework with other advanced DL techniques seeks to highlight its superiority. The model achieved accuracy scores of 93% for ultrasound scans and 95% for breast histopathology scans. Using DTL, Pati et al.^22^ created a model for a fully automated breast cancer diagnosis system that utilized data from the Cancer Imaging Archive. The accuracy of an SVM classifier prediction was enhanced using Transfer Learning methods. The experimental accuracy on a substantial mammography dataset comprising benign and malignant pictures was notably high at 97.99%, surpassing some of the monocentric research based on mammogram images. Almutairi et al.^23^ studied the features of newly generated BRCA images. After the feature extraction is completed, they segment the images using Caffe-Net and apply Improved Random Forest (IRF) XGBoost (XGB). The model achieved 97.87% accuracy for ultrasound images. The model achieved for mammography images 98.31% accuracy. Nemade et al. ^24^, enhanced the breast cancer diagnostic efficacy by the provision of two DL-based frameworks. To validate the suggested models, experiments using two separate mammography datasets are performed. As basic classifiers, VGG16, InceptionV3, and VGG19 (type of CNN classifier) were used. Two ensemble frameworks were developed to enhance the accuracy of breast lesion identification in mammographic pictures. The DDSM dataset produced Specificity, Accuracy, and Sensitivity values of 98.87%, 97.17%, as well as 98.02% for Ensemble Model 1. The corresponding results for Ensemble Model 2 were 97.01%, 98.10%, 99.12%, along with 97.01%.

Sachdeva et al.^25^ devised a systematic method of classifying BC by comparing four classifiers Wisconsin Breast Cancer Original (WBCO) dataset. When compared to other classifiers, the accuracy (98.31%) obtained by KNN with feature selection was the greatest. Rehman et al.^26^ emphasized the sub-types of breast cancer categorization. The DL classifiers were employed to train in the DL framework, and the ultrasound dataset was pre-processed to increase its quality. Two DL models, MobileNetV2 and DenseNet201, were used to compose the deep ensemble model in the proposed method. The proposed classification approach had a success rate of 97.04% in categorizing breast cancer pictures. Mohamed et al.^27^ suggested using the gene expression data from The Cancer Genome Atlas (TCGA), a bio-inspired CNN model was proposed for the detection of breast cancer. A comparison was conducted between the classification outcomes achieved by the suggested approach and those attained by the conventional CNN, along with five hybrid algorithms. High-performance measures were used to ascertain class membership, as well as the results indicated that the proposed framework attained a kappa of 90.3% with an accuracy of 98.3% for the malignant class. Table 2 shows the above literature studies in tabular format.

**Table 2:** Previous research done by various authors.

Authors	Technique used	Outcomes	Limitations
Zakareya et al.^21^	Automated DL model	Accuracy: 93% (ultrasound), 95% (histopathology)	Focuses mainly on accuracy without addressing interpretability or generalizability
Pati et al.^22^	Transfer Learning + SVM	Accuracy: 97.99%	Reliant on monocentric mammography datasets limiting broader applicability
Almutairi et al.^23^	Caffe-Net + XGBoost	Accuracy: 97.87% (ultrasound), 98.31% (mammography)	Computationally intensive, limiting real-time applicability
Nemade et al.^24^	Ensemble CNN	Accuracy: 97–98%, Sensitivity up to 99.12%	Ensemble increases complexity and is tested on limited mammography datasets
Sachdeva et al.^25^	KNN	Accuracy: 98.31%	Classical ML may underperform on complex image data compared to deep learning methods
Rehman et al.^26^	MobileNetV2 + DenseNet201	Classification success rate: 97.04%	Dependent on ultrasound image quality and uses a complex ensemble architecture
Mohamed et al.^27^	Bio-inspired CNN	Accuracy: 98.3%, Kappa: 90.3%	Limited to gene expression data, restricting imaging diagnosis and integration potential

## Problem statement

One of the most prevalent and deadly illnesses affecting women worldwide is breast cancer, for which early and precise detection is essential to successful treatment and higher survival rates. Despite advancements in medical imaging technologies, existing computer-aided diagnostic systems often fall short due to limitations in accuracy, speed, and resilience, making them prone to human error and delayed diagnoses. Interpreting complex medical images continues to pose challenges for radiologists, and current clinical decision support systems (CDSS) are often inadequate in addressing these complexities. As a result, there is a critical need for a highly advanced and intelligent diagnostic framework that integrates ensemble deep-learning techniques to enhance diagnostic precision, reduce uncertainty, and support timely medical decision-making.

## The proposed model

**Fig. 2:**
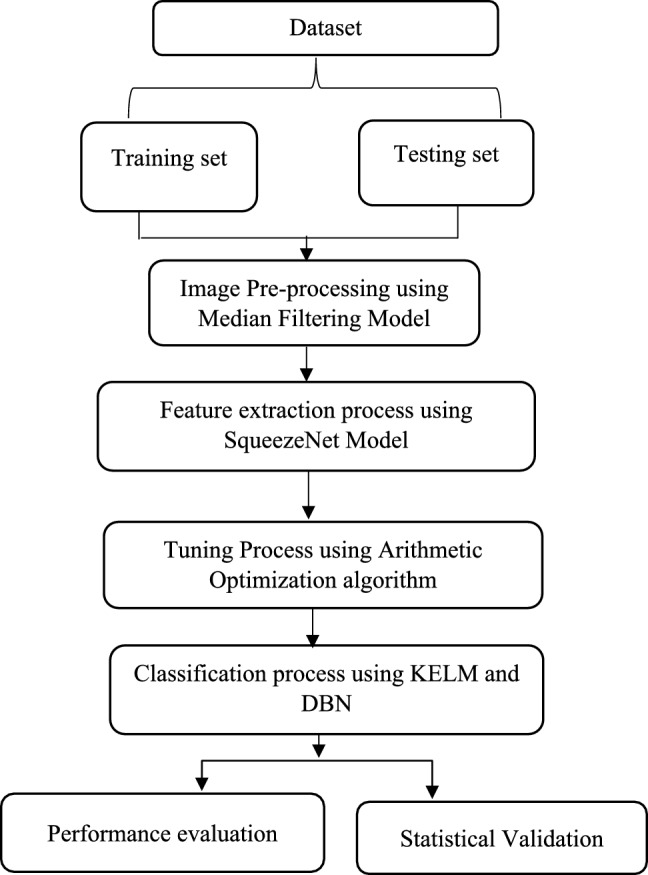
Proposed flowchart of EDL-CDSS.

Current research established an innovative EDL-CDSS method to determine the presence of breast cancer using a structured deep-learning pipeline. This method begins with the division of the dataset into training as well as testing sets, followed by an image pre-processing stage using a median filtering model to reduce noise and enhance image quality. Next, SqueezeNet is employed to extract meaningful features from the processed images, ensuring efficiency and high-level abstraction. These features are then optimized through the Arithmetic Optimization Algorithm, which tunes the hyperparameters of the classification models. Finally, classification is performed using an ensemble of KELM and DBN to categorize images as either positive or negative for breast cancer. The complete EDL-CDSS approach is visually represented in Fig. 2.

### Pre-processing using the median filter model

All image processing techniques commence with preprocessing. The primary objective of this method is to improve picture quality by reducing artifacts as well as distortions while emphasizing essential features for further processing. These photos need preprocessing because they are more challenging to understand than other types of medical images^28^. Preprocessing pictures and capitalizing on their inherent redundancy greatly improves the accuracy of optical inspections. In this study, images have been preprocessed using a median filter model^29^.

The concept of median filtering was invented by John Tucci, who also presented a technique of nonlinear signal processing^30^. This kind of separation allows for the edge of an image to maintain its clarity while simultaneously removing background noise. In this sort, adjacent pixels are evaluated based on their brightness levels, and the central value becomes the new reference for the focal pixel. This filter smooths the image without shifting the edges, which can occur with more traditional smoothing filters^31^.

### Feature extraction using ensemble model

A series of DL models is utilized during feature extraction Known as SqueezeNet.

#### SqueezeNet

**Fig. 3:**
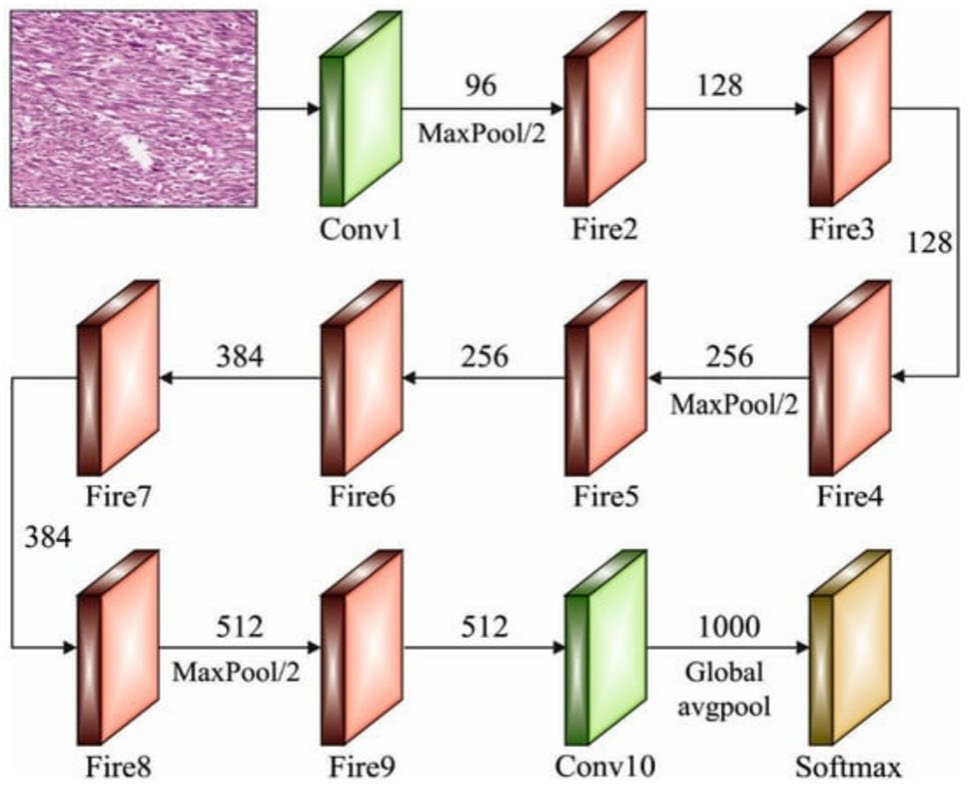
SqueezeNet architecture^34^.

SqueezeNets are a specific kind of Deep Neural Network (DNN) that typically have 18 layers and are used in information technology software. Designing a tiny NN with fewer parameters and facilitating transmission across a computer network were the key aims of the authors in the creation of SqueezeNet. Furthermore, it should have a low memory footprint on the machine. The original version of this framework was deployed using Caffe, a DL framework^32^. After a few amounts of time, the authors began implementing this framework in a variety of open-source DL architectures. First, SqueezeNet was given a label, after which it was put through a competition with AlexNet. Despite being distinct DNN frameworks, AlexNet and SqueezeNet have a common trait: a high degree of accuracy in predicting the ImageNet picture dataset. Figure 3 presents an illustration of the SqueezeNet architecture^33^.

SqueezeNet functions as the primary feature extractor in the ensemble pipeline by converting preprocessed ultrasound images into high-level abstract representations with minimal computational cost^34^. These extracted features are then passed to the classifiers DBN and KELM for decision-making. Unlike DBN, which performs hierarchical generative modeling, or KELM, which focuses on kernel-based discriminative classification, SqueezeNet uniquely contributes by efficiently encoding spatial and semantic image features. Its lightweight architecture ensures fast processing without sacrificing accuracy, thus enhancing the ensemble’s overall performance by providing rich, compact features that improve both training efficiency and prediction robustness across the model^34^.

### Tuning process using arithmetic optimization algorithm

The Arithmetic Optimization Algorithm (AOA) is a nature-inspired metaheuristic technique that uses basic arithmetic operations to efficiently explore and exploit the search space for optimization problems^35^. In the proposed EDL-CDSS framework, AOA is employed to tune hyperparameters of deep learning models like SqueezeNet, DBN, and KELM. It initializes a population of candidate hyperparameter sets and iteratively updates them by applying arithmetic operators probabilistically to balance global exploration and local exploitation. This process helps identify the optimal hyperparameter values that maximize classification accuracy, improving training efficiency and enhancing the overall diagnostic performance of the breast cancer detection system.

### Breast cancer image classification

At this point, the data that were removed from the outliers are input into the image classification model, which is comprised of two models and is referred to respectively as KELM and DBN. The following is a definition for each of these two approaches:

#### KELM model

ELM’s output function in the case of a single output node is:

1 $$f\left(x\right)=\sum_{i-1}^{L}{\beta }_{i}G\left({a}_{i},{b}_{i},x\right)=\beta .h\left(x\right)$$

When $\beta ={[{\beta }_{1},\dots ..{\beta }_{L}]}^{T}$ denotes the cumulative total weight. $G({a}_{i},{b}_{i},x)$ indicates that the result of the ith hidden layer, along with the node parameters, is generated randomly. *h(x)*=${[G\left({a}_{1},{b}_{1},x\right)\dots \dots .G({a}_{L},{b}_{L},x)]}^{T}$ denotes the result of the layer that is hidden as an expression of the input. The kernel function can thereafter be computed as^36,37^:

2 $${\Omega }_{ELM}={HH}^{T}:{\Omega }_{ELM}=h\left({x}_{i}\right).h\left({x}_{j}\right)=K({x}_{i}.{x}_{j})$$

**Fig. 4:**
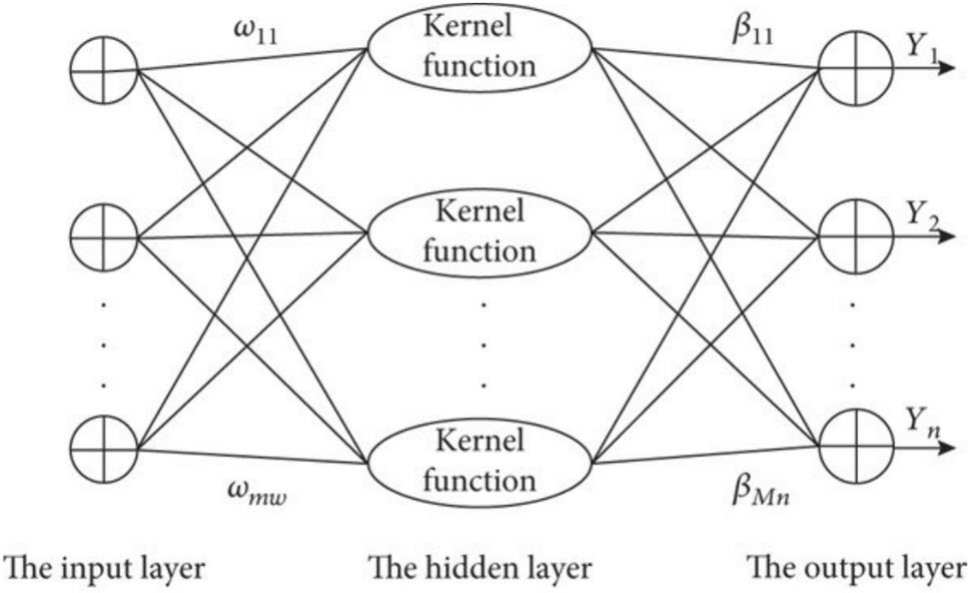
The structure chart of the KELM^40^.

ELM classifier’s output function is characterized by^38^

3 $$f\left(x\right)=h(x){H}^{T}{\left(\frac{1}{\lambda }+{HH}^{T}\right)}^{-1}T=\left[K\left(x,{x}_{1}\right) . . . K(x,{x}_{N}) \right]{\left(\frac{1}{\lambda }+{\Omega }_{ELM}\right)}^{-1}T$$

where $I$ is the identity matrix, $\lambda$ is the normalization coefficient, and $T$ is the label for the set that was trained^39^. After applying this model, the kernel function would be used for the final computation, not the precise form of the feature map h(x). Consequently, it is unnecessary to ascertain the extent of the concealed layer L, and it is possible to circumvent arbitrary biases and weights. Figure 4 illustrates the KELM structure.

#### Deep belief network (DBN)

DNN differs mainly in two ways from DBN:

- **Network topology:** As a feedforward network, a DNN has multiple hidden layers. Furthermore, each concealed neuron implements the logistic/sigmoid activation mechanism. On the other hand, the hidden layers of layered Restricted Boltzmann Machines (RBM) employed by the DBN are coupled randomly.
- **Network training:** The backpropagation learning of a deep neural network depends on labeled data to optimize its weight. Conversely, DBN does its first training in an unsupervised fashion via contrasting divergence, thereafter refining its weights via backpropagation.

**Fig. 5:**
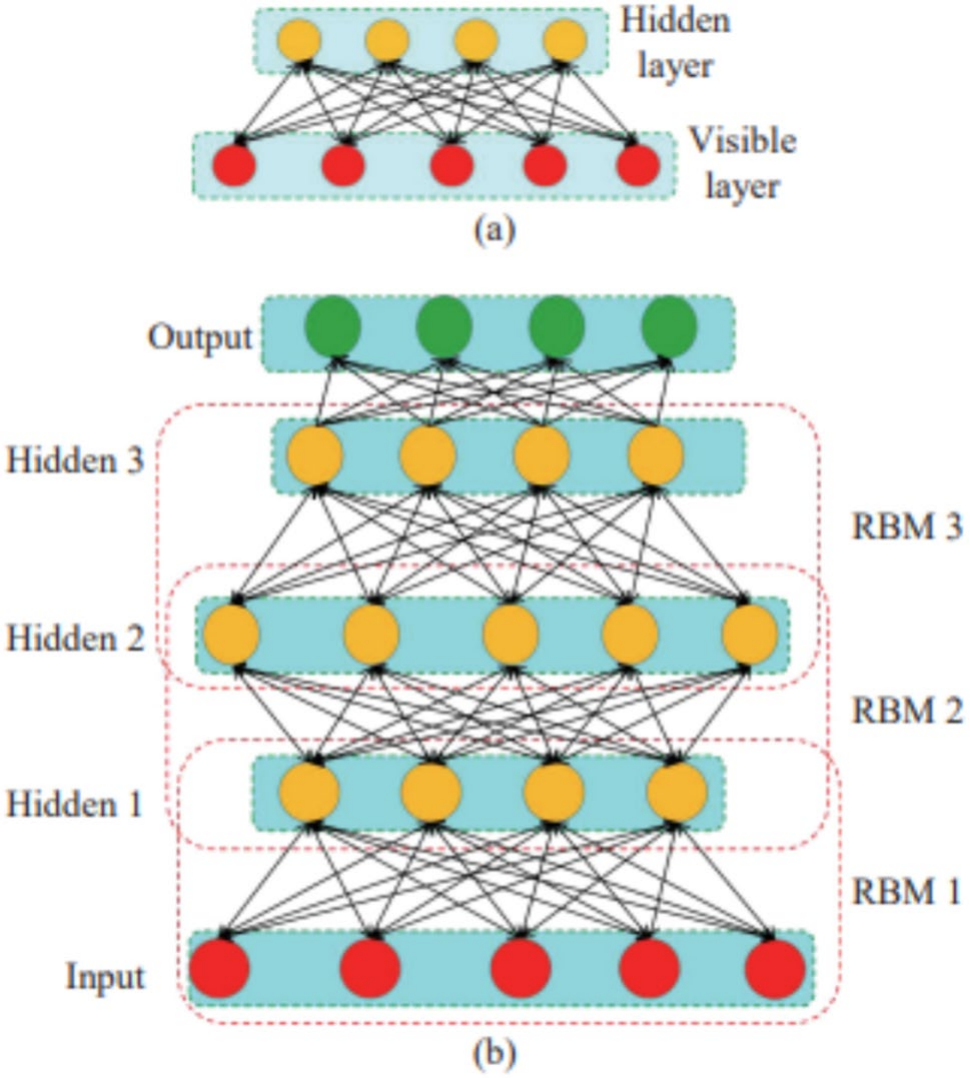
Structure of the DBN^45,46^.

A substantial number of equally distributed labels is required for DNN; however, most real-world datasets do not contain such labels. A Bayesian generative model, known as a DBN, is frequently constructed by layering RBMs. The stacked RBM’s parameters are adjusted using the CD technique^41^. The CD represents a form of unsupervised learning, thereby rendering labeled data unnecessary. The gradient descent learning procedure and an SL model, like SoftMax/LR or a linear classifier, would then be used to refine the previously learned network^42^. The DBN features are primarily defined immediately following the CD, while the second phase merely modifies the model’s attributes. As a result, DBN requires fewer tagging data^43^. A typical network organizational arrangement is shown in Fig. 5^44^.

## Hyperparameters tuning configuration

The performance of the proposed EDL-CDSS framework heavily relies on the careful tuning of hyperparameters across its core components: SqueezeNet, DBN, and KELM. To automate and optimize this process, the AOA is utilized to explore predefined search spaces for each hyperparameter, enabling the selection of values that maximize classification accuracy. The tuning covers a range of critical hyperparameters, including learning rates, dropout rates, number of training epochs, batch sizes, number of hidden units (for DBN), kernel width, and regularization parameters (for KELM), as shown in Table 3. Each hyperparameter is assigned a continuous or integer search range based on domain knowledge and empirical evidence. AOA operates with a fixed population size of 20 candidate solutions (agents), evolving over 50 generations. The fitness function guiding this evolutionary process is classification accuracy measured on validation data. By iteratively updating candidate hyperparameter sets using arithmetic operations, AOA efficiently balances exploration and exploitation, converging on optimal configurations that enhance model training efficiency and overall detection performance.

**Table 3:** Hyperparameter search space and settings for EDL-CDSS components.

Component	Hyperparameter	Search Space / Range	Type
SqueezeNet	Learning rate	0.0001 to 0.01	Continuous
	Dropout rate	0.3 to 0.7	Continuous
	Number of epochs	10 to 100	Integer
	Batch size	5 to 20	Integer
DBN	Number of hidden units	100 to 500	Integer
	Learning rate	0.001 to 0.1	Continuous
	Number of epochs	10 to 100	Integer
	Batch size	5 to 20	Integer
KELM	Kernel width (σ)	0.1 to 10	Continuous
	Regularization parameter (C)	0.01 to 100	Continuous
AOA Settings	Population size	20 agents	Fixed
	Number of generations	50	Fixed
	Fitness function	Classification Accuracy	Objective

The hyperparameters selected through the AOA in this study were optimized specifically for the Breast Ultrasound Images Dataset from Kaggle. This dataset includes grayscale images with specific imaging characteristics such as resolution, noise patterns, and lesion contrast, all of which influence model performance and the effectiveness of certain hyperparameter configurations. However, the core structure of ultrasound imaging, especially in breast diagnostics, tends to share common patterns across datasets (e.g., lesion texture, edge gradients, and background tissue consistency). Therefore, the tuned hyperparameters (such as the SqueezeNet learning rate, DBN hidden units, or KELM kernel width) are likely to provide a strong baseline when applied to similar ultrasound datasets.

## Evaluation metrics

The authors evaluated the suggested segmentation method using measures including Jaccard score, Specificity, Dice roll, and Accuracy within the context of picture segmentation^47^.

Equation 4 demonstrates that the Jaccard similarity index is applicable for comparing the ground-truth picture with the segmented image derived from the proposed segmentation technique 7. The resemblance between the segmented as well as ground-truth pictures is noticeable when the Jaccard and Dice coefficients are elevated.

4 $$Jaccard \left(M,N\right)=\frac{|M\cap N|}{\left|M\right|+\left|N\right|-|M\cap N|}$$

Equation 5 demonstrates that Dice is the appropriate similarity measure to evaluate how well the obtained segmented picture matches the ground truth.

5 $$Dice=2\frac{|M\cap N|}{|M|+|N|}$$

where M is the dataset’s accessible ground truth, and N is the segmented picture obtained using the suggested segmentation method.

**Accuracy:** Accuracy is a measure of the model’s general accuracy; it is the percentage of all occurrences that are properly predicted, positive and negative included.

6 $$\text{Accuracy}=(\text{TP}+\text{TN})/(\text{TP}+\text{TN}+\text{FP}+\text{FN})$$

**Precision:** A model’s precision, also referred to as its positive prediction value, indicates how accurate its forecasts are.

7 $$Precision=TP/(TP+FP)$$

**Recall:** Recall is a measure of how well the model detected real positives; it is sometimes called sensitivity or the true positive rate.

8 $$Recall=TP/(TP+FN)$$

**F1-Score:** Precision and Recall are harmonically averaged to get the F1-Score. When classes are unbalanced, it helps to balance the trade-off between recall and precision.

9 $$F1 Score=2\times (Precision\times Recall)/(Precision+Recall)$$

## Dataset description

The Breast Ultrasound Images Dataset used in this research, sourced from https://www.kaggle.com/datasets/aryashah2k/breast-ultrasound-images-dataset/data, consists of 1,578 grayscale ultrasound images categorized into three classes: normal (266 images), malignant (421 images), and benign (891 images), as shown in Table 4. The Breast Ultrasound Dataset is publicly available, IRB-exempt, and contains no patient identifiers, ensuring compliance with ethical standards. Each image is in PNG format, with ground truth binary masks available for benign and malignant classes to support tumor localization, while normal images lack associated masks. For this study, the dataset was divided into 80% training (1,260 images) and 20% testing (318 images). This dataset underpins the EDL-CDSS framework, enabling efficient classification via KELM and DBN models and allowing for statistical validation to enhance breast cancer diagnostic decision support.

**Table 4:** Dataset description.

Class	Training samples	Testing samples	Total samples	Class
Benign	712	179	891	Benign
Malignant	336	85	421	Malignant
Normal	212	54	266	Normal

## Experimental validation

**Fig. 6:**
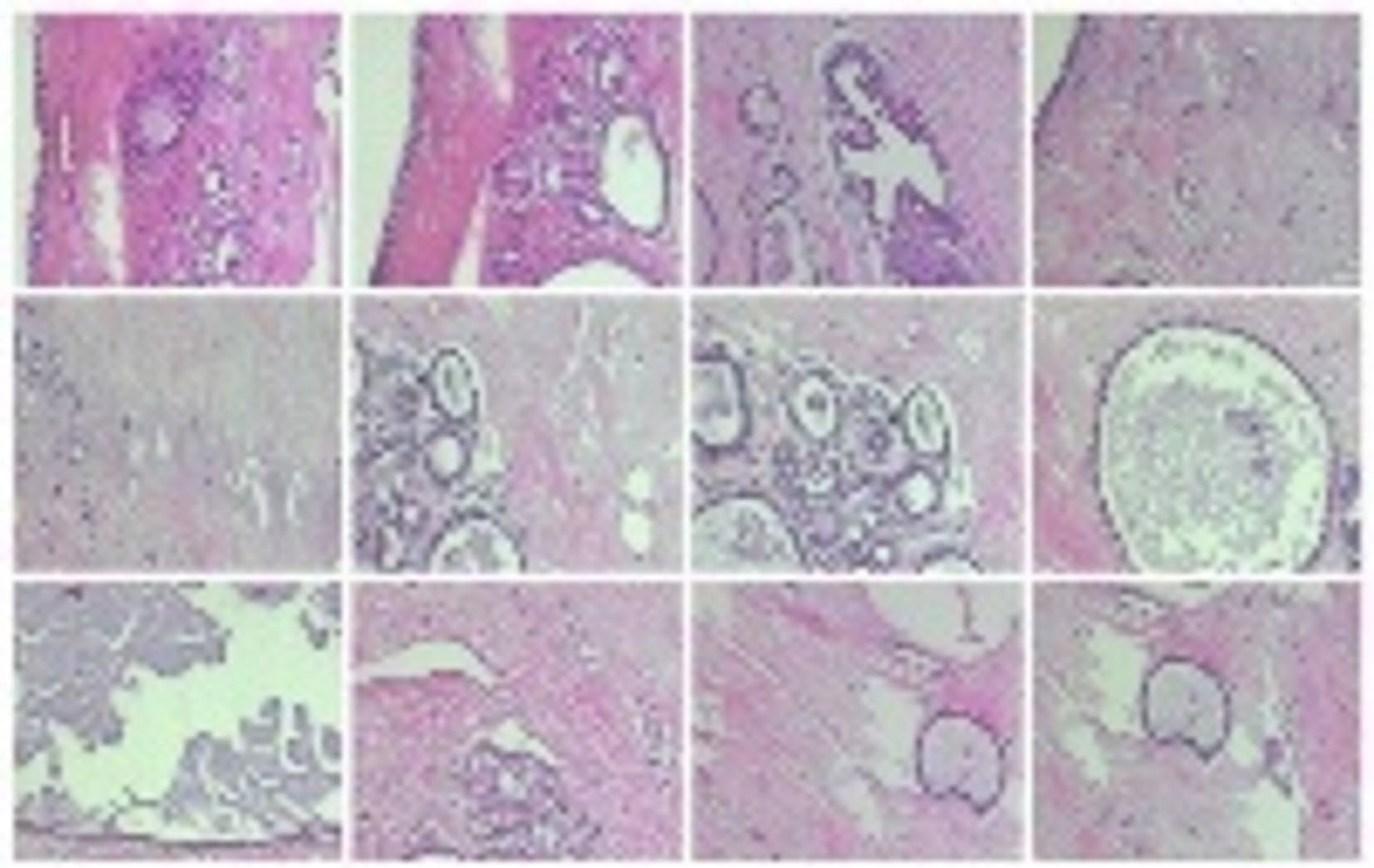
Sample images.

**Fig. 7:**
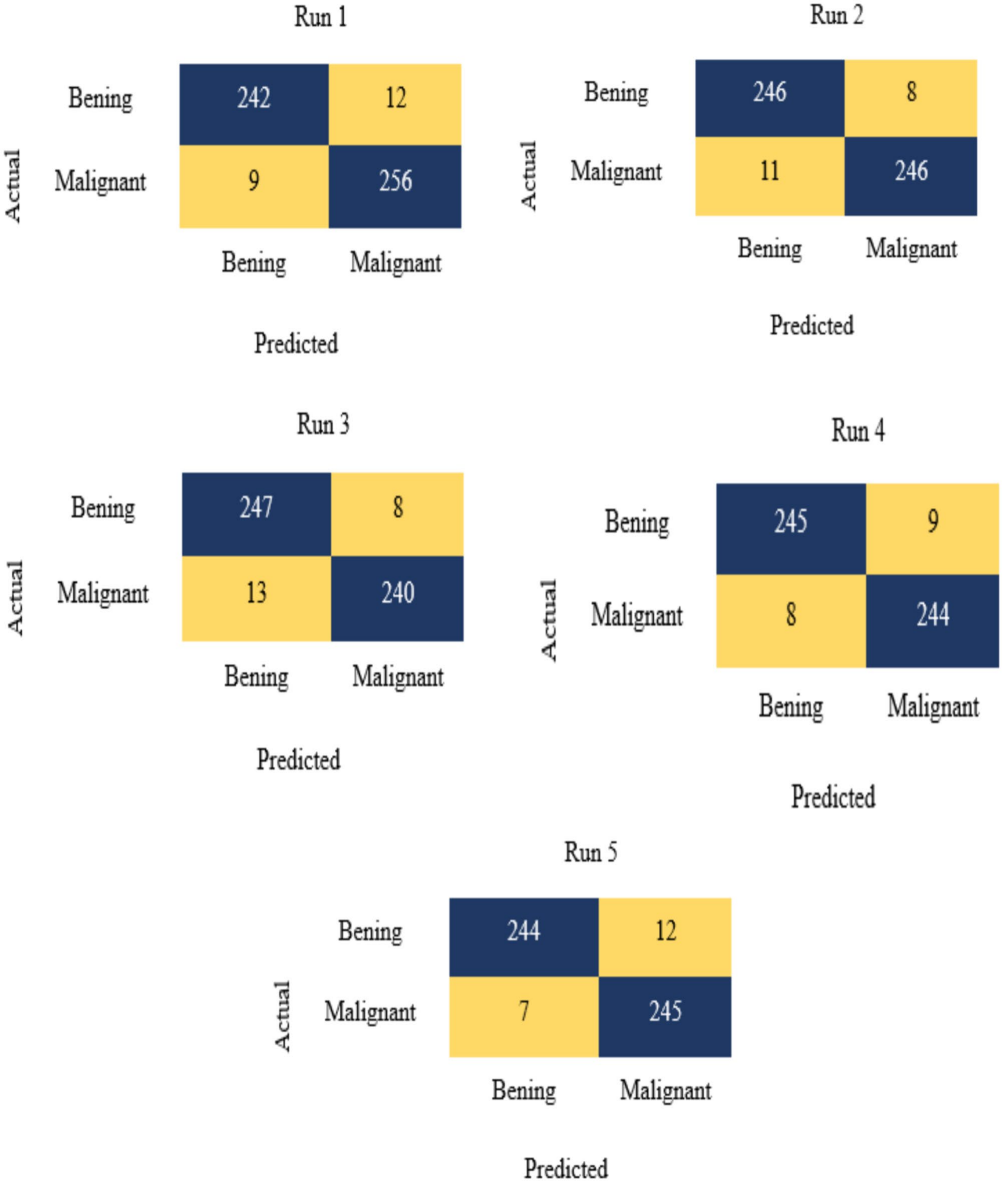
Confusion matrix analysis of the proposed method with different runs.

In this part, the findings of the EDL-CDSS model’s categorization of breast cancer on a benchmark dataset are analyzed and discussed. Figure 6 displays a sample from the dataset, and Fig. 7 displays the confusion matrices that were created by applying the proposed method to the test benchmark Breast Ultrasound Dataset data under five different runs. Many performance measures have been examined for the classification of benign as well as cancer images, such as Accuracy, Precision, F1-score, Kappa, and Recall, for both binary classifications. For both binary classifications, these metrics have been implemented.

Python 3.6.5 was used to simulate the suggested model on a PC with an i5-8600 k and a GeForce 1050Ti with 4 GB of video memory. The following values were specified for the parameters: Dropout = 0.5, batch size = 5, epoch count = 50, and activation = ReLU; learning rate = 0.01. Table 5 shows the evaluation based on measurements of the EDL-CDSS tests’ findings from each of five successive runs.

**Table 5:** Measurement-based evaluation of EDL-CDSS results from five separate runs.

No. of runs	Accuracy	Recall	Precision	F1-score	Kappa	Jaccard	Dice
Run-1	95.9	95.2	96.4	95.7	96.43	95.36	97.36
Run-2	96.2	96.8	95.7	96.2	95.71	96.27	95.61
Run-3	95.8	96.8	95	95.8	96.19	96.04	95.72
Run-4	96.6	96.4	96.8	96.5	95.8	95.81	95.05
Run-5	96.2	95.3	97.2	96.2	95.58	96.43	96.45
Average	96.14	96.08	96.22	96.1	95.8	95.9	96.03

**Fig. 8:**
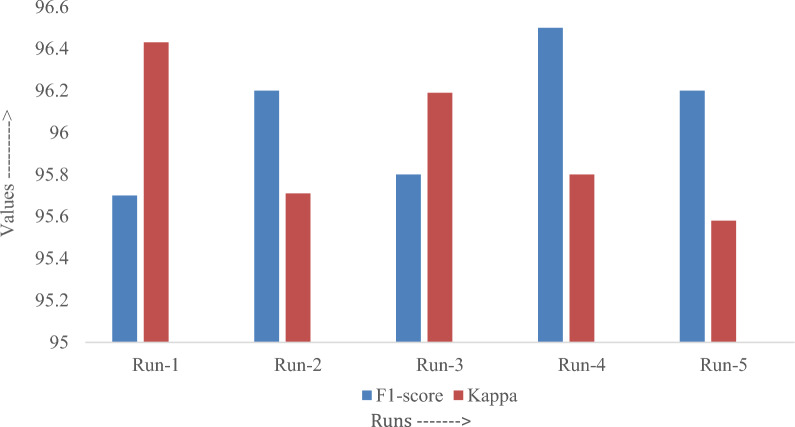
F1-score and Kappa analysis of the proposed method with different runs.

Figure 8 illustrates the F1-score as well as the Kappa analysis of the EDL-CDSS approach. The investigation was executed over five distinct test runs. The EDL-CDSS approach attained an F1-score of 95.7, along with a Kappa of 96.43 in run-1. These scores are in accordance with the standard. In addition, the method that was suggested produced an F1-score and a Kappa of 96.2 and 95.71, respectively, when applied to run-2. The proposed technique attained an F1-score as well as Kappa values of 96.5 and 95.8, respectively, in run 4.

**Fig. 9:**
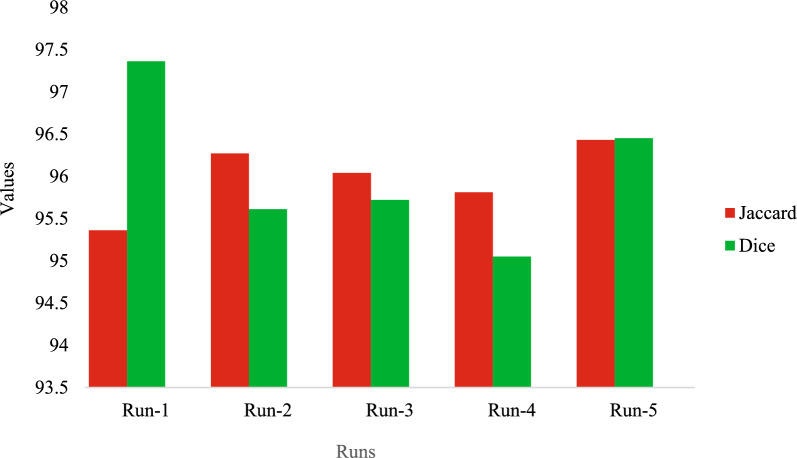
Jaccard and dice analysis of the suggested method with different runs.

Figure 9 displays the outcomes of the Jaccard as well as the Dice analysis of the proposed system over five distinct test runs. It was mentioned in the figure that the strategy that was provided had improved both the Jaccard and Dice values. For example, the strategy that was proposed obtained Jaccard and Dice scores of 95.36 and 97.36, respectively with run-1. Moreover, the proposed strategy has yielded Jaccard and Dice coefficients of 96.27 and 95.61 correspondingly, by applying run-2. Such numbers were found to be right. Finally, applying run-5 gave Jaccard and Dice indices of 96.43 and 96.45, respectively, as achieved in the suggested method.

**Fig. 10:**
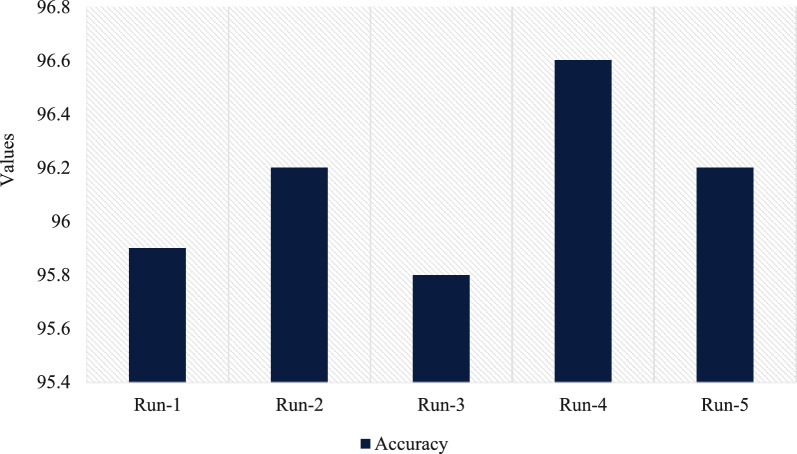
Proposed technique accuracy analysis with various runs.

Using the approach that was proposed, an assessment of the accuracy analysis that was done on the test dataset is shown in Fig. 10. The findings indicate that the proposed method was successful in improving dataset classification performance, as shown by the highest accuracy scores of 95.9, 96.2, 95.8, 96.6 and 96.2 for each of the five test runs, respectively.

**Fig. 11:**
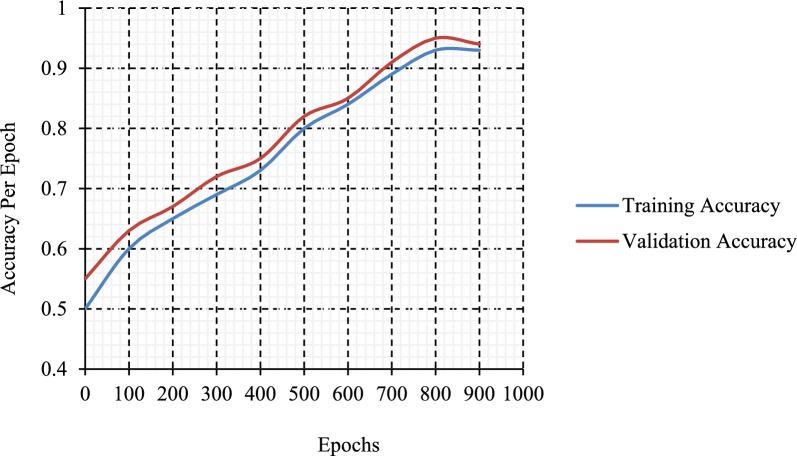
Accuracy graph analysis of the suggested method.

**Fig. 12:**
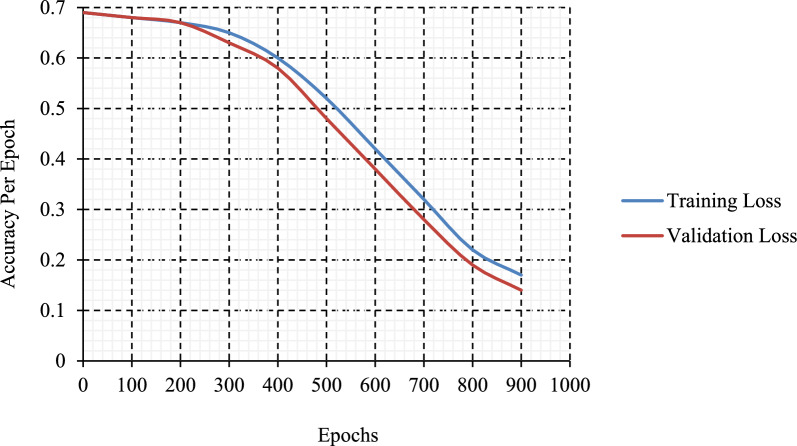
Loss graph analysis of the proposed method.

Figure 11 illustrates the accuracy analysis of the test dataset using the suggested methodology. The results indicated that the efficacy of the suggested technique improved with the augmentation of validation as well as training accuracy. This strategy achieved greater validation accuracy, as seen in Fig. 12, which presents the loss analysis findings on the test dataset under the proposed methodology. The outcomes indicated that the suggested strategy yielded a proficient output while minimizing training and validation loss. The proposed system has achieved a lower validation loss relative to the training loss.

## K-fold cross validation

This classification report, as shown in Table 6, presents the performance metrics of the DBN model used for breast cancer detection. The model achieved an overall accuracy of 89% on 318 test images. The class-wise performance showed precision, recall, and F1-scores of 0.90, 0.92, 0.91 for Benign (179 images), 0.89, 0.86, 0.87 for Malignant (85 images), and 0.83 for all three metrics in the Normal class (54 images). The macro average and weighted average F1-scores were both 0.87 and 0.89, respectively, reflecting strong and balanced performance.

**Table 6:** Deep belief network (DBN) classification report.

Class	Precision	Recall	F1-Score	Support
0	0.90	0.92	0.91	179
1	0.89	0.86	0.87	85
2	0.83	0.83	0.83	54
Accuracy			0.89	318
Macro Average	0.87	0.87	0.87	318
Weighted Average	0.89	0.89	0.89	318

This classification report, as shown in Table 7, presents the performance metrics of the KELM model. The KELM classifier achieved its best performance, resulting in an overall accuracy of 91.51%. This report summarizes the classifier’s performance across three classes: Class 0 (Benign), Class 1 (Malignant), and Class 2 (Normal). Notably, the model attained the highest recall (0.96) and F1-score (0.93) for Class 0, indicating strong sensitivity in identifying benign cases. Class 1 (Malignant) showed a precision of 0.96, reflecting excellent positive predictive value. The macro average and weighted average metrics further confirm the model’s well-rounded performance, highlighting its effectiveness and balance in handling imbalanced class distributions.

**Table 7:** KELM classification report.

Class	Precision	Recall	F1-score	Support
0	0.90	0.96	0.93	179
1	0.96	0.86	0.91	85
2	0.90	0.85	0.88	54
Accuracy			0.92	318
Macro Average	0.92	0.89	0.90	318
Weighted Average	0.92	0.92	0.91	318

The SqueezeNet classification report in Table 8 shows that the model performs well across three classes with an overall accuracy of 87.4%. Precision ranges from 85.2 to 88.9%, recall varies between 81.5% and 91.3%, and the F1-score, which balances precision and recall, lies between 84.7% and 90.1%. The support indicates roughly equal sample sizes for each class (around 330 instances each). The macro and weighted averages of precision, recall, and F1-score are all around 87.4%, reflecting consistent performance across classes and accounting for class imbalance. Overall, the model demonstrates solid and balanced classification capability across all categories.

**Table 8:** SqueezeNet classification report.

Class	Precision	Recall	F1-score	Support
0	0.8810	0.8155	0.8470	336
1	0.8522	0.8936	0.8724	329
2	0.8895	0.9134	0.9013	335
Accuracy			0.8740	1000
Macro Average	0.8742	0.8742	0.8736	1000
Weighted Average	0.8744	0.8740	0.8736	1000

Table 9 of the ensemble model combining SqueezeNet and KELM achieves a high accuracy of 96.23%, indicating excellent classification performance overall. Precision across classes ranges from 95 to 99%, showing the model’s ability to correctly identify positive instances. Recall varies between 93 and 98%, reflecting strong sensitivity to detecting actual positives. The F1 scores, balancing precision and recall, range from 94 to 97%, indicating robust classification across all classes. The support indicates an imbalanced dataset, with class 0 having the most samples (179) and class 2 the least (54). Macro and weighted averages of precision, recall, and F1-score are all around 96%, demonstrating consistent and reliable performance across classes, accounting for the class distribution.

**Table 9:** Ensemble model classification report.

Class	Precision	Recall	F1-score	Support
0	0.95	0.98	0.97	179
1	0.99	0.94	0.96	85
2	0.96	0.93	0.94	54
Accuracy			0.9623	318
Macro Average	0.97	0.95	0.96	318
Weighted Average	0.96	0.96	0.96	318

Table 10 presents the average performance metrics for four models: KELM, DBN, SqueezeNet, and their Ensemble (SqueezeNet + KELM). The Ensemble model shows the highest mean accuracy (96.03%), precision (96.10%), recall (96.03%), specificity (96.11%), and F1-score (96.01%), indicating superior overall classification ability. Among individual models, KELM performs better than DBN and SqueezeNet across all metrics, with an accuracy of around 89.76%. DBN shows the lowest average performance with accuracy near 79.37%. SqueezeNet’s performance lies between DBN and KELM, with an accuracy of approximately 87.40%. These mean values provide a clear comparison of the typical effectiveness of each technique.

**Table 10:** Mean of various parameters for different methods.

Technique	Accuracy	Precision	Recall	Specificity	F1-score
KELM	0.8976	0.8994	0.8976	0.9036	0.8968
DBN	0.7937	0.7945	0.7937	0.8497	0.7933
SqueezeNet	0.8740	0.8768	0.8740	0.9372	0.8731
Ensemble (SqueezeNet + KELM)	0.9603	0.9610	0.9603	0.9611	0.9601

Table 11 shows the variability (standard deviation**)** of the performance metrics for the same four models. Lower values indicate more consistent results across trials or folds. The Ensemble model exhibits near-zero standard deviations for accuracy, precision, and recall (close to zero, e.g., 1.24e−16), demonstrating highly stable and reliable performance. KELM also has low variability, with standard deviations generally under 0.01. DBN shows the highest variability, particularly in accuracy and recall (about 0.017), indicating less stable results. SqueezeNet’s variability is moderate, with values around 0.013–0.014. These std values highlight how consistent the models’ performances are in different runs or data splits.

**Table 11:** Standard deviation of various parameters for different methods.

Technique	Accuracy	Precision	Recall	Specificity	F1-score
KELM	0.0063	0.0064	0.0063	0.0102	0.0063
DBN	0.0172	0.0160	0.0172	0.0147	0.0166
SqueezeNet	0.0139	0.0127	0.0139	0.0068	0.0146
Ensemble (SqueezeNet + KELM)	1.24e−16	7.44e−04	1.24e−16	0.0065	1.89e−04

## Component contribution and hyperparameter optimization analysis

Table 12 evaluates the contribution of individual pipeline components, median filtering, arithmetic optimization, and preprocessing by training and testing each classifier (SqueezeNet, DBN, and KELM) separately on the same dataset. By systematically removing one component at a time, the impact on accuracy was measured. The accuracy remains constant at 0.8850 for all classifiers across all variations, indicating that these preprocessing and optimization components do not significantly affect the model performance individually. This suggests that the classifiers themselves drive the majority of the predictive power, and the pipeline components tested are not critical for accuracy. Additionally, hyperparameter tuning yielded optimized parameter values (5.0089, 0.0790) with a very low loss of approximately 0.00087. This result highlights that fine-tuning model parameters is an important factor in achieving optimal performance, potentially more impactful than the removal or inclusion of the tested preprocessing steps. Together, these findings emphasize focusing efforts on classifier architecture and hyperparameter optimization rather than adjustments to median filtering, arithmetic optimization, or preprocessing to improve model performance effectively.

**Table 12:** Component contribution of various techniques.

Technique	Full pipeline	No median filter	No arithmetic optimization	No preprocessing
SqueezeNet	0.8850	0.8850	0.8850	0.8850
DBN	0.8850	0.8850	0.8850	0.8850
KELM	0.8850	0.8850	0.8850	0.8850

## Statistical validation

Table 13 illustrates the classification accuracy scores achieved by four different models, Proposed, KELM, DBN, and SqueezeNet, across five experimental runs. The Proposed model consistently exhibits the highest performance, with accuracies ranging from 95.30 to 96.42%. KELM shows stable performance, around 93.91% across all runs, indicating reliable but moderate performance. DBN has a slight variability but remains competitive. SqueezeNet maintains a consistent but comparatively lower accuracy of 93.04% across all runs, highlighting its limited predictive capability relative to the other models.

**Table 13:** Classification accuracy across five experimental runs.

Model	Run 1	Run 2	Run 3	Run 4	Run 5
Proposed	0.9581	0.9642	0.9587	0.9601	0.9530
KELM	0.9391	0.9391	0.9391	0.9391	0.9391
DBN	0.9391	0.9370	0.9413	0.9377	0.9391
SqueezeNet	0.9304	0.9304	0.9304	0.9304	0.9304

The statistical evaluation using paired t-tests and one-way ANOVA in Table 14 demonstrates that the Proposed EDL-CDSS model significantly outperforms the baseline models in terms of classification accuracy. The paired t-test results show statistically significant differences between the Proposed model and KELM (t = 10.8713, p = 0.0004), DBN (t = 8.2197, p = 0.0012), and SqueezeNet (t = 15.6756, p = 0.0001), indicating that the Proposed model consistently delivers higher performance. Additionally, the one-way ANOVA test (F = 129.6369, p = 0.0000) confirms that there is a significant variation in accuracies across all the models compared. These findings validate the effectiveness and robustness of the Proposed model in breast cancer classification tasks.

**Table 14:** Statistical comparison of proposed model with baseline models.

Comparison	Test type	t/F Value	p-value	Significance
Proposed vs KELM	Paired t-test	10.8713	0.0004	Significant (✓)
Proposed vs DBN	Paired t-test	8.2197	0.0012	Significant (✓)
Proposed vs SqueezeNet	Paired t-test	15.6756	0.0001	Highly Significant (✓)
All Models (Proposed, KELM, DBN, SqueezeNet)	One-way ANOVA	129.6369	0.0000	Significant (✓)

**Fig. 13:**
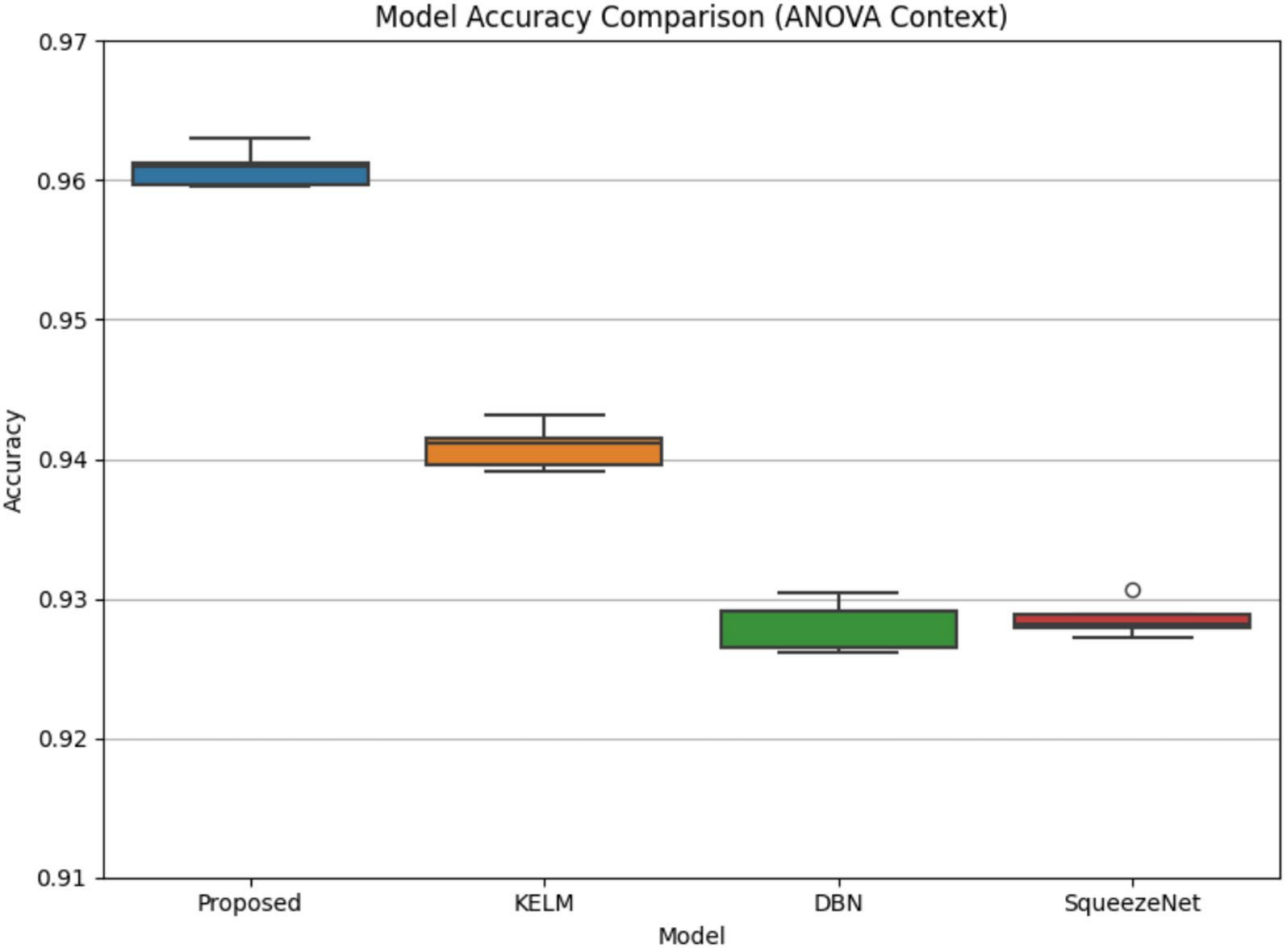
Model accuracy comparison (ANOVA context).

Figure 13 visualizes the distribution of classification accuracies for different models across multiple experimental runs in the context of ANOVA. The Proposed model exhibits the highest accuracy distribution, centered tightly around 0.96, with minimal variance, indicating both high performance and consistency. KELM and DBN models show moderate performance, with DBN having a wider interquartile range (IQR), reflecting higher variability in its results. SqueezeNet has the lowest median accuracy and displays an outlier, further emphasizing its inferior and less stable performance. Overall, the figure visually supports the statistical findings that the Proposed model significantly outperforms the others in both accuracy and consistency.

## Comparison analysis

**Fig. 14:**
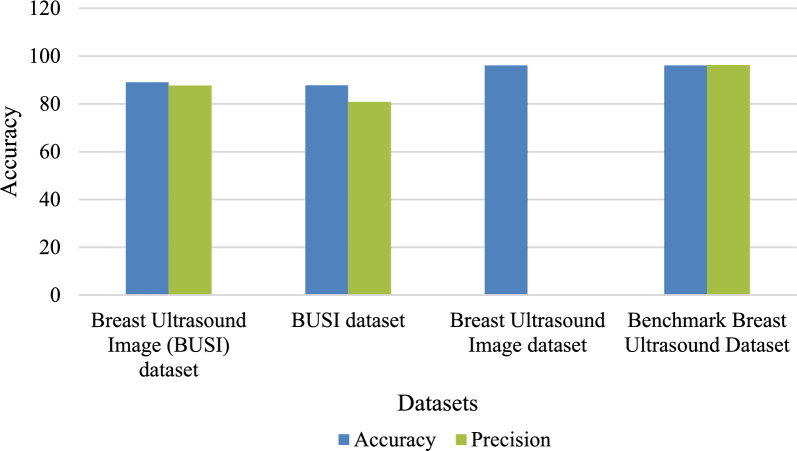
Evaluation of the new method against the existing methods.

**Fig. 15:**
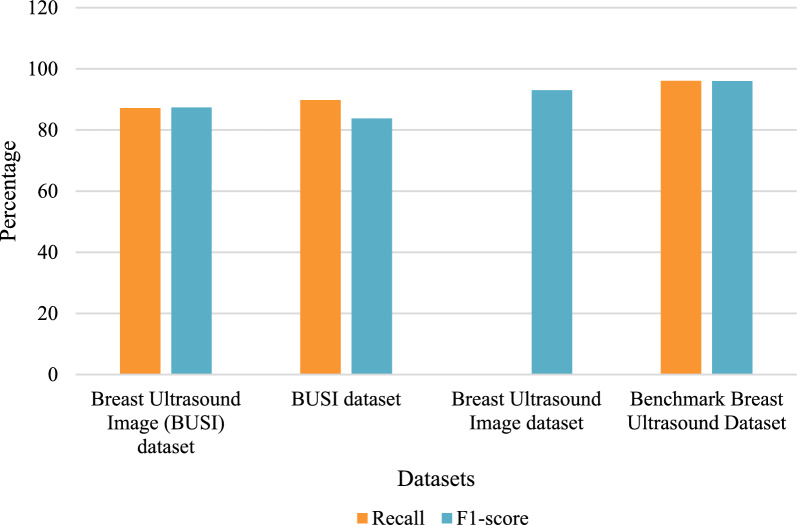
Comparison of the suggested method recall and F1-score with other approaches.

Figure 14 presents a comprehensive study that compares the accuracy and precision of the EDL-CDSS algorithm with those of other algorithms already in use. According to the figure, the Breast Ultrasound Image (BUSI) dataset has achieved a less-than-optimal level of performance, with an accuracy of 87.8% and precision of 80.80%. However, the projected suggested system has shown the other methods with a greater accuracy of 96.14% and precision of 96.22%. Figure 15 shows the comprehensive study that compares the F1-score and recall of the proposed method with others. Table 15 shows the effectiveness of a variety of classifiers over many datasets.

**Table 15:** Analyzing the performance of different classifiers.

Authors [References]	Dataset Used	Accuracy	Precision	Recall	F1-score
Abhisheka et al.^48^	Breast Ultrasound Image (BUSI) dataset	89.02	87.67	87.17	87.36
Alotaibi et al.^49^	BUSI dataset	87.8	80.8	89.8	83.8
Joshi et al.^50^	Breast Ultrasound Image dataset	96.10	–	–	92.99
Proposed model	Benchmark Breast Ultrasound Dataset	96.14	96.22	96.08	96.01

## Conclusion

A precise and timely diagnosis is crucial for obtaining the appropriate treatment, as breast cancer has a high incidence and a high likelihood of fatality. New technology, notably DL algorithms, has demonstrated positive outcomes in improving the precision of breast cancer diagnosis. In this investigation, the development of EDL-CDSS is introduced. This technique was devised to improve the precision and dependability of breast cancer detection. The EDL-CDSS employs many DL architectures, including KELM and DBN, to identify intricate patterns in medical imaging data. Comprehensive evaluations are conducted on baseline data sets, and the outcomes are juxtaposed with the latest advanced methodologies to evaluate the efficacy of the EDL-CDSS. This indicates that the approach has a superior success rate, underscoring its effectiveness as a crucial instrument for the early identification of breast cancer. The experimental outcomes demonstrate that the proposed model exhibits superior F1-score, Recall, Precision, and Accuracy at 96.1%, 96.08%, 96.22%, and 96.14% respectively, compared to the alternatives.

### Ethical and data statements

The proposed EDL-CDSS framework demonstrates strong potential for clinical translation and real-world applicability in breast cancer diagnostics. By leveraging lightweight deep learning models like SqueezeNet along with robust classifiers such as KELM and DBN, the system offers high accuracy with low computational overhead—making it suitable for integration into existing clinical imaging workflows. Its ability to deliver reliable and rapid diagnostic support can assist radiologists in early breast cancer detection, particularly in resource-constrained settings where expert interpretation may be limited. The incorporation of statistical validation further ensures the system’s reliability and generalizability, enhancing its readiness for deployment in real-world medical environments.

## Data Availability

https://www.kaggle.com/datasets/aryashah2k/breast-ultrasound-images-dataset/data.

## References

[1] Kaushal, C., Koundal, D. & Singla, A. Comparative analysis of segmentation techniques using histopathological images of breast cancer. In *2019 3rd International Conference on Computing Methodologies and Communication (ICCMC)*, 261–266 (2019).

[2] Mohapatra, S. K., Jain, A. & Sahu, P. Comparative approaches by using machine learning algorithms in breast cancer prediction. In *2022, 2nd International Conference on Advance Computing and Innovative Technologies in Engineering (ICACITE)* 2022, 1874–1878.

[3] Masud, M. et al. Pre-trained convolutional neural networks for breast cancer detection using ultrasound images. *ACM Trans. Internet Technol.* **21**(4), 1–17 (2021).

[4] Thigpen, D., Kappler, A. & Brem, R. The role of ultrasound in screening dense breasts—A review of the literature and practical solutions for implementation. *Diagnostics* **8**(1), 20 (2018). 29547532 10.3390/diagnostics8010020 PMC5872003

[5] Kumar, S. S., Radhakrishnan, A. K. & Cheong, S. K. Rapid metastasis of breast cancer cells from a primary tumor to liver. *Pak. J. Biol. Sci.* **13**(7), 303–315 (2010). 20836285 10.3923/pjbs.2010.303.315

[6] Idris, N. F. & Ismail, M. A. Breast cancer disease classification using fuzzy-ID3 algorithm with FUZZYDBD method: Automatic fuzzy database definition. *PeerJ Comput. Sci.* **7**, e427 (2021). 34013024 10.7717/peerj-cs.427 PMC8114800

[7] Preetha, R. & Jinny, S. V. A research on breast cancer prediction using data mining techniques. *Int. J. Innov. Technol. Explor. Eng.* **8**(11S2), 362–370 (2019).

[8] Adam, R., Dell’Aquila, K., Hodges, L., Maldjian, T. & Duong, T. Q. DL applications to breast cancer detection by magnetic resonance imaging: A literature review. *Breast Cancer Res.* **25**, 87 (2023). 37488621 10.1186/s13058-023-01687-4 PMC10367400

[9] Assiri, A. S., Nazir, S. & Velastin, S. A. Breast tumor classification using an ensemble machine learning method. *J. Imaging* **6**(6), 39 (2020). 34460585 10.3390/jimaging6060039 PMC8321060

[10] Dehghan Rouzi, M. et al. Breast cancer detection with an ensemble of DL networks using a consensus-adaptive weighting method. *J. Imaging* **9**(11), 247 (2023). 37998094 10.3390/jimaging9110247 PMC10671922

[11] Shen, L. et al. DL to improve breast cancer detection on screening mammography. *Sci. Rep.* **9**(1), 12495 (2019). 31467326 10.1038/s41598-019-48995-4 PMC6715802

[12] Rautela, K., Kumar, D. & Kumar, V. A systematic review on breast cancer detection using DL techniques. *Arch. Comput. Methods Eng.* **29**(7), 4599–4629 (2022).

[13] Bhushan, A., Gonsalves, A. & Menon, J. U. Current state of breast cancer diagnosis, treatment, and theranostics. *Pharmaceutics* **13**(5), 723 (2021). 34069059 10.3390/pharmaceutics13050723 PMC8156889

[14] Yala, A., Lehman, C., Schuster, T., Portnoi, T. & Barzilay, R. A. DL mammography-based model for improved breast cancer risk prediction. *Radiology* **292**(1), 60–66 (2019). 31063083 10.1148/radiol.2019182716

[15] Iacopetta, D., Ceramella, J., Baldino, N., Sinicropi, M. S. & Catalano, A. Targeting breast cancer: An overlook on current strategies. *Int. J. Mol. Sci.* **24**(4), 3643 (2023). 36835056 10.3390/ijms24043643 PMC9959993

[16] Greenwood, H. I. et al. Ductal carcinoma in situ of the breasts: A review of MR imaging features. *Radiographics* **33**(6), 1569–1588 (2013). 24108552 10.1148/rg.336125055

[17] Zuluaga-Gomez, J., Zerhouni, N., Al Masry, Z., Devalland, C. & Varnier, C. A survey of breast cancer screening techniques: Thermography and electrical impedance tomography. *J. Med. Eng. Technol.* **43**(5), 305–322 (2019). 31545114 10.1080/03091902.2019.1664672

[18] Krawczyk, B., Schaefer, G. & Zhu, S. Y. Breast cancer identification based on thermal analysis and a clustering and selection classification ensemble. In *Brain and Health Informatics: International Conference (BHI)* Maebashi, Japan, October 29–31, 256–265 (2013).

[19] Hassan, A. M. & El-Shenawee, M. Review of electromagnetic techniques for breast cancer detection. *IEEE Rev. Biomed. Eng.* **4**, 103–118 (2011). 22273794 10.1109/RBME.2011.2169780

[20] Patel, B. K. et al. MR elastography of the breast: Evolution of technique, case examples, and future directions. *Clin. Breast Cancer* **21**(1), e102–e111 (2021). 32900617 10.1016/j.clbc.2020.08.005 PMC8486355

[21] Zakareya, S., Izadkhah, H. & Karimpour, J. A. New deep-learning-based model for breast cancer diagnosis from medical images. *Diagnostics* **13**(11), 1944 (2023). 37296796 10.3390/diagnostics13111944 PMC10253109

[22] Pati, A. et al. Breast cancer diagnosis based on IoT and deep transfer learning enabled by fog computing. *Diagnostics* **13**(13), 2191 (2023). 37443585 10.3390/diagnostics13132191 PMC10340497

[23] Almutairi, S. M. et al. An efficient USE-Net DL model for cancer detection. *Int. J. Intell. Syst.* **2023**, 8509433 (2023).

[24] Nemade, V., Pathak, S. & Dubey, A. K. Deep learning-based ensemble model for classification of breast cancer. *Microsyst. Technol.* **2023**, 1–15 (2023).

[25] Sachdeva, R. K., Bathla, P., Rani, P., Kukreja, V. & Ahuja, R. Systematic method for breast cancer classification using RFE feature selection. In *Proceedings of the 2022 2nd International Conference on Advance Computing and Innovative Technologies in Engineering (ICACITE)*, Greater Noida, India, 28–29 April 2022, 1673–1676 (2022).

[26] Rehman, M. Z. U. et al. An efficient automated technique for classification of breast cancer using deep ensemble model. *Comput. Syst. Sci. Eng.* **46**(1), 897–911 (2023).

[27] Mohamed, T. I., Ezugwu, A. E., Fonou-Dombeu, J. V., Ikotun, A. M. & Mohammed, M. A bio-inspired convolution neural network architecture for automatic breast cancer detection and classification using RNA-Seq gene expression data. *Sci. Rep.* **13**(1), 14644 (2023). 37670037 10.1038/s41598-023-41731-z PMC10480180

[28] Ramani, R., Vanitha, N. S. & Valarmathy, S. The pre-processing techniques for breast cancer detection in mammography images. *IJ Image Graph. Signal Process.* **5**, 47–54 (2013).

[29] George, M. J. & Dhas, D. A. S. Preprocessing filters for mammogram images: A review. In *2017 Conference on Emerging Devices and Smart Systems (ICEDSS)*, 1–7 (IEEE, 2017).

[30] Vorobyev, N. V. CIfroviye komporatory. Prodolzheniye [Digital Comparators. Sequel] Journal Chip News. 1999. №7.

[31] Bulyaculov, R. R., Schogoleva, K. P., Yakovlev, I. N. & Roskostov, R. A. Modelling and analysis of the median filter algorithm of suppression of impulse noise. In *Proceedings of the 2017 IEEE Conference of Russian Young Researchers in Electrical and Electronic Engineering (EIConRus)*, St. Petersburg and Moscow, Russia, 01–03 February 2017, 649–654 (2017).

[32] Muhammad, Y., Alshehri, M. D., Alenazy, W. M., Vinh Hoang, T. & Alturki, R. Identification of pneumonia disease applying an intelligent computational framework based on DL and machine learning techniques. *Mobile Inf. Syst.* **2021**, 1–20 (2021).

[33] Hilal, A. M. et al. An Intelligent DL based hyperspectral Signal classification scheme for complex measurement systems. *Measurement* **188**, 110540 (2022).

[34] Fakieh, B., Al-Ghamdi, A. S. A. M. & Ragab, M. Optimal deep stacked sparse autoencoder based osteosarcoma detection and classification model. *Healthcare* **10**(6), 1040 (2022). 35742091 10.3390/healthcare10061040 PMC9222514

[35] Basheri, M. Intelligent breast mass classification approach using archimedes optimization algorithm with DL on digital mammograms. *Biomimetics* **8**(6), 463 (2023). 37887593 10.3390/biomimetics8060463 PMC10604039

[36] Roushangar, K., Alirezazadeh Sadaghiani, A. & Shahnazi, S. Novel application of robust GWO-KELM model in predicting discharge coefficient of radial gates: A field data-based analysis. *J. Hydroinf.* **25**(2), 275–299 (2023).

[37] Fan, Q., Meng, X., Nguyen, D. T., Xie, Y. & Yu, J. Predicting displacement of bridge based on CEEMDAN-KELM model using GNSS monitoring data. *J. Appl. Geod.* **14**(3), 253–261 (2020).

[38] Li, C., Zhou, J., Dias, D. & Gui, Y. A kernel extreme learning machine-grey wolf optimizer (KELM-GWO) model to predict the uniaxial compressive strength of rock. *Appl. Sci.* **12**(17), 8468 (2022).

[39] Li, J., Hai, C., Feng, Z. & Li, G. A transformer fault diagnosis method based on parameters optimization of hybrid kernel extreme learning machine. *IEEE Access* **9**, 126891–126902 (2021).

[40] Cao, L., Yue, Y. & Zhang, Y. A novel fault diagnosis strategy for heterogeneous wireless sensor networks. *J. Sens.* **2021**, 1–18 (2021).

[41] Hinton, G. E., Osindero, S. & Teh, Y. W. A fast learning algorithm for deep belief nets. *Neural Comput.* **18**(7), 1527–1554 (2006). 16764513 10.1162/neco.2006.18.7.1527

[42] Deep Learning 0.1 documentation: Deep Belief Networks. http://deeplearning.net/tutorial/DBN.html

[43] Feng, W., Wu, S., Li, X. & Kunkle, K. A deep belief network-based machine learning system for risky host detection. arXiv preprint 2017, arXiv:1801.00025.

[44] AlKhateeb, J. H. & Alseid, M. DBN-Based learning for Arabic handwritten digit recognition using DCT features. In Proceedings of the *2014 6th International Conference on Computer Science and Information Technology (CSIT)*, Amman, Jordan, 26–27 March 2014, 222–226 (2014).

[45] Chen, X. M. et al. Design and analysis for early warning of rotor UAV based on data-driven DBN. *Electronics* **8**(11), 1350 (2019).

[46] Xiong, D., Zhang, D., Zhao, X. & Zhao, Y. DL for EMG-based human-machine interaction: A review. *IEEE/CAA J. Autom. Sin.* **8**(3), 512–533 (2021).

[47] Kaushal, C., Bhat, S., Koundal, D. & Singla, A. Recent trends in computer-assisted diagnosis (CAD) system for breast cancer diagnosis using histopathological images. *IRBM* **40**(4), 211–227 (2019).

[48] Abhisheka, B., Biswas, S. K., Purkayastha, B. & Das, S. Integrating deep and handcrafted features for enhanced decision-making assistance in breast cancer diagnosis on ultrasound images. *Multimed. Tools. Appl.* **2025**, 1–26 (2023).

[49] Alotaibi, M. et al. Breast cancer classification based on convolutional neural network and image fusion approaches using ultrasound images. *Heliyon* **9**(11), e22406 (2023). 38074874 10.1016/j.heliyon.2023.e22406 PMC10700613

[50] Joshi, R. C., Singh, D., Tiwari, V. & Dutta, M. K. An efficient deep neural network based abnormality detection and multi-class breast tumor classification. *Multimed. Tools Appl.* **81**(10), 13691–13711 (2022).

